# Provision and use of physical rehabilitation services for adults with disabilities in Rwanda: A descriptive study

**DOI:** 10.4102/ajod.v11i0.1004

**Published:** 2022-08-30

**Authors:** Anne Kumurenzi, Julie Richardson, Lehana Thabane, Jeanne Kagwiza, Ines Musabyemariya, Jackie Bosch

**Affiliations:** 1 Department of Rehabilitation Sciences, Faculty of Health Sciences, McMaster University, Hamilton, Canada; 2 Department of Physiotherapy, Faculty of Health Sciences, University of Rwanda, Kigali, Rwanda; 3 Department of Health Research Methods, Evidence, and Impact, Faculty of Health Sciences, McMaster University, Hamilton, Canada; 4 Population Health Research Institute, McMaster University, Hamilton, Canada; 5 Functional Rehabilitation Programme, Humanity and Inclusion, Kigali, Rwanda

**Keywords:** adults, disabilities, physical rehabilitation, outpatient services, health facilities, descriptive study, Rwanda

## Abstract

**Background:**

Physical rehabilitation interventions address functional deficits caused by impairments that affect someone’s performance. Whilst rehabilitation is important, it is assumed that these services are either minimal or nonexistent in low-resource settings. Our data expand on the data from the Situation Assessment of Rehabilitation in the Republic of Rwanda report to describe rehabilitation services and who access them at public and semiprivate facilities (primarily funded by the private sector).

**Objectives:**

This article describes the use of the outpatient physical rehabilitation services across nine health facilities, the characteristics of adults attending these health facilities and some of the facilitators and barriers they encounter when attending rehabilitation.

**Method:**

Data were collected between September and December 2018 from the heads of departments and adult patients attending outpatient rehabilitation services funded by the government, international nongovernmental organisations or faith-based organisations.

**Results:**

Two hundred and thirteen adults were recruited from nine facilities. There is a sixfold difference in the number of rehabilitation personnel between public and semiprivate hospitals in these facilities’ catchment areas. However, most participants were recruited at public facilities (186 [87%]), primarily with physical disorders. Patients reported that family support (94%) was the most crucial facilitator for attending rehabilitation, whilst transportation cost (96%) was a significant barrier.

**Conclusion:**

Rehabilitation service availability for Rwandan adults with disabilities is limited. Whilst family support helps patients attend rehabilitation, transportation costs remain a significant barrier to people attending rehabilitation. Strategies to address these issues include developing triage protocols, training community health workers and families.

**Contribution:**

Data on rehabilitation service provision in Rwanda and most African countries are either non-existent or very limited. These data contain important information regarding the services provided and the people who used them across different health facilities (public versus private) and urban versus rural settings). To improve rehabilitation service provision, we first need to understand the current situation. These data are an important step to better understanding rehabilitation in Rwanda.

## Introduction

Adult physical functioning is affected by a variety of health conditions, such as noncommunicable diseases (NCDs) or injuries (Manini 2012). Noncommunicable diseases are increasing in prevalence worldwide and particularly in low- and middle-income countries (Gimigliano & Negrini 2017; World Health Organization [WHO] 2017). Despite the growing burden of physical disabilities in low- and middle-income countries (Jesus, Landry & Hoenig 2019; Jesus et al. 2021), which more than doubled from 1990 to 2017 in low-income countries (Jesus et al. 2019), the provision of rehabilitation is limited in many low- and middle-income countries (Bright, Wallace & Kuper 2018). Physical rehabilitation interventions optimise function and minimise physical disability for those whose physical impairments affect performance (Bowker et al. 2006, 2011, 2017), with the intent to equip people to live to their maximum potential and optimise their contribution to family, community and society (Wade 2020; Bowker et al. 2006). In high-income countries, physical rehabilitation is usually provided by credentialed, skilled health professionals, such as but not limited to rehabilitation physicians, psychologists, physiotherapist (PTs), occupational therapist (OTs), speech and language therapists (SLTs), prosthetists and orthotists (P&Os) and nurses. Despite the importance of these services, the demand for these services in many low- and middle-income countries exceeds their human resources, leading to continued poor functional outcomes (Bright et al. 2018; Jesus et al. 2019; Jesus et al. 2021; Prynn & Kuper 2019). As a result, the demand for these services exceeds the resources (Bright & Kuper 2018; Bright et al. 2018; Prynn & Kuper 2019), leading to continued poor functional outcomes.

In response to the growing need for rehabilitation, in 2017, the World Health Organization (WHO) launched the Rehabilitation 2030 initiative. This initiative brought together an international group of key stakeholders in rehabilitation service provision to develop strategies and action plans to provide quality and timely rehabilitation worldwide, particularly in low- and middle-income countries with limited rehabilitation services and the greatest need (Gimigliano & Negrini 2017). These countries were urged to collect data on rehabilitation needs and services and the accessibility and availability of services to guide the efficient implementation of quality services (WHO 2017). Evidence on disability and rehabilitation data is an important first step in understanding the needs and gaps in rehabilitation service provision to develop efficient solutions (Gimigliano & Negrini 2017; WHO 2014).

Rwanda is a low-income country in central east Africa with 13.4 million people, which has been projected to increase to 16.9 million by 2023. Life expectancy is 70 years for women and 66 years for men (Vollset et al. 2020). According to a 2012 census, 5% of Rwandans aged 5 years or older have a disability, in which 80% of those with a disability are 18 years or older, almost 90% live in rural settings and 25% report mobility limitations (walking or climbing) (National Institute of Statistics of Rwanda & Ministry of Finance and Economic Planning-Rwanda 2014). In a more recent 2019–2020 survey, 14% of Rwandans aged 5 years or older reported at least one functional limitation, in which 69% of those with a functional limitation are 18 years or older (National Institute of Statistics of Rwanda Kigali, Rwanda Ministry of Health Kigali, Rwanda & The DHS Program ICF 2021). Therefore, the number of adults in Rwanda with disabilities seems to be increasing.

Rwandan disability rates are similar to estimates from other African countries (e.g., 7.5% in South Africa in 2011) (Statistics South Africa 2014), but much lower than the Westernised rates (e.g., 22.0% in Canada in 2017) (Morris et al. 2018). Whilst both the Rwandan and South African estimates indicate there are fewer persons with disabilities, it is likely that both these estimates are inaccurate. There is a greater stigma associated with disability in African countries (Loeb 2013; M’Kumbuzi et al. 2014); therefore, persons with disabilities may be reluctant to self-identify themselves as having a disability (Loeb 2013). With increasing life expectancy and prevalence of NCDs in Rwanda, it is quite possible that actual prevalence rates for disability are at least as high as Western countries (Alleyne et al. 2013; Asiimwe-Kateera et al. 2015; Tapela et al. 2015).

In 2007, Rwanda put in effect Law no. 01/2007 of 20 January 2007 relating to the protection of persons with disabilities, with the intent of creating more inclusivity for person with disability (Rwandan Ministry of Justice 2009). However, the law was based on a medical model of disability, limiting the understanding of disability to the issues associated with the person and ignoring the role of structures (e.g., environment, systems). In 2021, the Ministry of Local Government issued the National Policy of Persons with Disability and Four Years Strategic Plan (2021–2024).^1^ This policy is based on a social model of disability to indicate a shift in thinking towards disability in Rwanda. Whilst the plan is a big step in the right direction, it focuses on children with disability. The increasing prevalence of NCDs in Rwanda (Alleyne et al. 2013; Asiimwe-Kateera et al. 2015; Tapela et al. 2015) and the resultant disability are not a consideration. Rehabilitation services for adults with disabilities in Rwanda may begin in the acute hospital setting; however, a large number of patients are likely to be discharged without rehabilitation (Rhoda et al. 2015). This is regardless of the level of disability. To further add to the problem, community-based services are extremely limited. To adequately and efficiently address the rehabilitation needs of those with a disability in Rwanda, we first need to better understand more accurate and comprehensive estimates of the prevalence of disability in Rwanda to predict rehabilitation resource needs accurately.

In Rwanda, the healthcare sector is organised around national referral or university teaching hospitals, provincial referral hospitals, district hospitals, primary health centres (PHCs) and health posts (Figure 1-A1). Physical rehabilitation services are available at public and private hospitals and at semiprivate specialised rehabilitation centres. Semiprivate facilities are funded primarily by the international non-governmental organisations (INGOs), such as Humanity & Inclusion (HI) and International Committee of Red Cross (ICRC), or faith-based organisations (FBOs), such as Christian Blind Mission. For more than 2.86 million Rwandans with a disability, publicly funded rehabilitation services are available at 53 hospitals: (1) 4 national referral or teaching, (2) 7 provincial or referral and (3) 42 district hospitals. In addition, those who can pay for specialised physical rehabilitation services are available at four semiprivate rehabilitation centres (Figure 1-A1; Ministry of Health 2018).

About half of Rwanda’s population is over the age of 18 years (7 208 063 out of 13 411 153 people).^2^ Therefore, suppose we conservatively estimate that 15% of Rwandan adults have a disability. In that case, roughly 1 000 000 Rwandan adults have access to 57 centres (including those not publicly funded) for rehabilitation interventions or approximately 17 500 persons with disabilities per centre.

Physiotherapy (PT) services are available at 53 hospitals and the 4 semiprivate specialised rehabilitation centres. According to the Rwanda Allied Health Professional Council (RAHPC), 360 PTs are registered to practice in Rwanda (Table 1), 88 of whom are employed at public facilities (Ministry of Health-Rwanda 2021), whilst the others likely work in private hospitals or INGOs. In 2019, there were 0.26 PTs per 10 000 people in Rwanda (WCPT 2019b), compared with 1.4 PTs per 10 000 people in South Africa (WCPT 2019a) and 6.6 PTs per 10 000 people in Canada (Canada Conference Board 2017). The number of PTs in Rwanda is far less than in other countries. Prosthetic and orthotic services are available at four public hospitals and at four specialised rehabilitation centres. According to the RAHPC, 68 P&Os are registered to practice, with 31 employed primarily in settings supported by INGOs. There are 0.05 P&Os per 10 000 people, which is precisely the minimum of 5 per one million (0.05 per 10 000 people) suggested by the Global Standards for Prosthetics and Orthotics (Lemaire, Supan & Ortiz 2018).

**TABLE 1a:** Distribution of rehabilitation services by funding source and type of health facility.

Funding source	Type of facility	Total no.* of facilities in Rwanda	Sample for the current study	Facilities sampled	No† of staff			Catchment area	Rehab Staff per 10 000 in catchment
					PTs†	P&Os†	OTs†		
Public hospitals (some private funding but primarily government†)	Tertiary National referral university teaching hospitals	4	2	Kigali University Teaching Hospital	9	4	0	750 000	0.2
				Rwanda Military Hospital	12	0	2	750 000	0.2
	District hospitals	42	3	Kabgayi Hospital	2	0	0	200 000	0.1
				Murunda Hospital	2	0	0	150 000	0.1
				Masaka Hospital	3	0	0	200 000	0.2
	Total	46	5	-	28	4	2	2 050 000	0.2
Semiprivate facilities (some government funding but primarily private)	Specialised hospitals	2	1	Inkurunziza Orthopedic Hospital	6	2	0	100 000	0.8
	Semiprivate rehab centres	4	3	Gahini Rehab Centre	9	3	5	150 000	1.1
				Rilima Rehab Centre	7	1	0	80 000	1.0
				Gatagara Rehab Centre	13	6	1	100 000	2.0
	Total	6	4	-	35	12	6	430 000	1.2
**Total (public and semiprivate)**	-	**48**	**9**	**-**	**63**	**16**	**8**	**2 480 000**	**0.4**

No., number; MOH, Ministry of Health; HI, Humanity & Inclusion; PTs, physiotherapists; P&Os, prosthetists and orthotists; OTs, occupational therapists. Catchment area is the area from which patients at the public hospital are drawn considering only the adult population and private facilities, the proportion within the area who can pay the required fees.

† , This is an estimate of those who would need rehabilitation services in the area and does not consider the number who could pay for the services. Therefore, the number of people who can access semiprivate services is considerably less.

The occupational therapy programme began at the University of Rwanda in 2014, and there are now occupational therapists (OTs) at one public hospital and two specialised rehabilitation centres. Twenty-six OTs are practicing in Rwanda, with most OTs (*n* = 24) employed in settings supported by INGOs, such as schools and refugee camps. There are 0.02 OTs per 10 000 Rwandans, which is lower than the minimum of 750 per one million (7.5 per 10 000 people) suggested by the World Federation of Occupational Therapists (WHO 2017). Speech and language therapists are nearly absent and available at only one private tertiary hospital and one semiprivate specialised rehabilitation centre. There are no speech language therapy programmes in the country. Representative of most African countries, Rwanda does not have rehabilitation services at the PHCs (Ministry of Health-Rwanda 2021).

In 2021, the Rwandan Ministry of Health (MoH), in collaboration with the WHO, the United States Agency International Development and the ICRC published a report on the Situation Assessment of Rehabilitation in the Republic of Rwanda. This report describes the insufficient and nonexistent rehabilitation services in Rwanda (Ministry of Health-Rwanda 2021). However, prior to this report, the MoH has proposed a new employment health structure that will integrate two PTs per PHC, one P&O per district hospital and one OT and one SLT employed at each referral and provincial hospital (Official Gazette 2020) for the first time. However, the progress of implementing the new employment health structure is taking time.

In addition to a lack of funding for rehabilitation service provision, there are issues unique to low- and middle-income countries compounding difficulties in accessing services. These include transportation expenses because of long travel distances to rehabilitation facilities, long waiting times for appointments and limited knowledge of the available services, both by the public and private personnel (Aenishänslin, Amara & Magnusson 2020; Baart & Taaka 2018; Hamid et al. 2017; Rhoda et al. 2015; Zziwa et al. 2019). The four semiprivate specialised rehabilitation centres in Rwanda are intended as a national resource for all individuals; however, there is an additional cost for these services, which further limits access (Ministry of Health-Rwanda 2021). These facilities have well-established rehabilitation services for children; however, few are tailored to adults (Ministry of Health-Rwanda 2021). As a result, adults with complex needs such as stroke, traumatic brain injuries and spinal cord injuries are discharged from acute wards to attend outpatient rehabilitation at a district hospital or in the community, where these services are either insufficient or nonexistent.

With limited human resources to provide rehabilitation interventions, it is imperative to allocate existing rehabilitation resources based on the population’s needs. The data available on the Rwandan needs for healthcare services are primarily on access to healthcare services for children and those with mental health issues (Hategeka, Arsenault & Kruk 2020; Rugema et al. 2015; Ng & Harerimana 2016; Smith et al. 2017; Wanyana, Wong & Hakizimana 2021). The lack of data on rehabilitation needs of adults with disabilities, despite a growing need because of increased survival rates for those with NCDs (cardiovascular diseases [hypertension, stroke], diabetes, cancer) (Alleyne et al. 2013; Asiimwe-Kateera et al. 2015; Tapela et al. 2015), makes efficient resource allocation impossible.

To address the lack of these data, HI, an INGO that provides rehabilitation services for adults and children in Rwanda, conducted a survey to understand costs associated with the provision of outpatient rehabilitation services. Using the data from HI on adults accessing rehabilitation services, we conducted a secondary data analysis to describe the type of rehabilitation services provided, the characteristics of people who use these services and the facilitators and barriers for people to access these services.

This article describes the outpatient physical rehabilitation services at nine facilities in Rwanda. We describe the rehabilitation services by funding source (public or semiprivate) and geographical locations (urban or rural). Finally, we use patient-level data to describe the people that use these services and some patients’ facilitators and barriers to attending rehabilitation.

## Research methods and design

### Study design

Humanity & Inclusion developed, funded and implemented a cross-sectional survey to understand the cost of rehabilitation services in Rwanda. From September to December 2018, in collaboration with the MoH and the National Commission of Persons with Disabilities, HI conducted the survey across 12 rehabilitation facilities supported by public and INGOs or FBOs in both urban and rural settings. However, this article presents data from nine facilities amongst adults with disabilities.

### Setting

The 12 health facilities that provide rehabilitation services were purposively selected. These facilities were selected based on the representation of levels of the health system pyramid (Figure 1-A1), two public university teaching hospitals, one public provincial hospital, two public district hospitals and four semiprivate rehabilitation centres. Facilities were also selected if they offered one of the physical rehabilitation services (PT, P&O, OT and SLT) and were supported by HI (funding to support service implementation for private facilities and provision of equipment for public facilities). This article includes data from only 9 facilities (as presented in Figure 1), in which the remaining 3 of the 12 facilities are specialised for children only.

**FIGURE 1:**
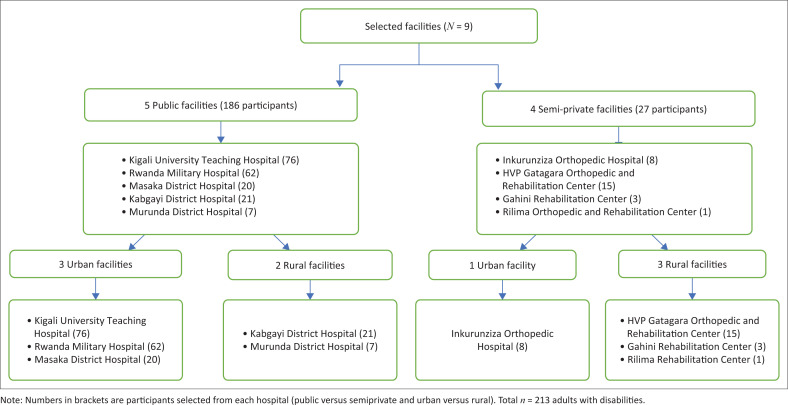
The number of recruited participants at each facility, divided into public versus semi-private facilities and urban versus rural areas.

### Study population

From September to December 2018, children and adults with disabilities were randomly selected by site champions across the 12 selected health facilities. All persons with disabilities or caregivers or parents or caregivers of children under 18 years old who agreed to participate and were present when data collectors were at the health facilities were recruited. Of the 385 participants recruited across 12 facilities, this article focuses on the data obtained from 213 adult respondents (18 years or older) with disabilities across 9 facilities. Data on service provision were obtained from the heads of departments.

To calculate the overall sample size required across the facilities to answer questions on cost-effectiveness, the Cochran’s formula was used as follows:


$$N=\frac{Z^{2}*p(1-p)}{d^{2}}$$ [Eqn 1]

(where *z* = z-score for the standard normal distribution at the significance level of 95%, *p* = anticipated proportion of people having access to rehabilitation service (50%) and δ = desired precision [5%]):


$$N=\frac{1.96^{2}*0.50(1-0.50)}{0.50^{2}}=385\text{ participants}$$ [Eqn 2]

Data were collected on 385 participants; however, data for this article included only the adults with disabilities who completed the survey (*n* = 213 participants) across 9 facilities.

### Data collection

The Improved Financial Access to Rehabilitation Services Diagnostics Tool (iFAR), a tool developed for use in low- and middle-income countries (Beguin & Boisgillot 2016; Boisgillot 2020), provides comprehensive data on the financial status of facilities that provide physical rehabilitation services. It is a 110-item survey that HI developed to understand the rehabilitation services provided and facility’s financial viability. It was completed by the heads of departments at each selected facility and by the eligible participants with disabilities attending rehabilitation services at these facilities. This tool was adapted for use in Rwanda by experts in disability and rehabilitation (Boisgillot & Umuhoza 2020), and 70 out of the 110 item points were used for this analysis. In addition to the data collected using iFAR, they collected patient-level data (i.e., patient’s sex, age, marital status, employment status, type of disability, level of education).

Two health economists (one from Rwanda) and two Rwandan PTs hired by HI coordinated data collection. Participants were recruited by HI research staff between September and December 2018, who were on site three days a week during that period. Data were collected by eight trained rehabilitation professionals using paper-based questionnaires and tablets (where feasible). All data were collected using lists with responses to select from or yes or no responses.

The data collectors participated in a three day training programme that included training on administration of the questionnaire, data collection procedures (recruitment, sampling) and ethical norms in research, particularly data collection. Each selected site had a site champion who was briefed on the type of participants needed for the study. Site champions randomly recruited all participants, and those who agreed to participate were referred to persons administering the survey. Eligible participants who were available during the three days of data collection, and agreed to participate, were recruited for the study. The data from the facilities and patients are presented by the source of funding (public or semiprivate) and geographical location (urban or rural). We were interested in assessing the differences between patients with disabilities who attended public and semiprivate facilities in urban and rural areas. Therefore, the data are presented in two sample proportions.

### Data analysis

Descriptive analyses are reported as counts, proportions for all variables and *p*-values for demographic characteristics. All analyses were performed using MS-Excel version 16 and STATA/IC 16 programmes.

## Results

Data were collected from nine facilities; five (56%) were publicly funded, and four (44%) were semiprivately funded. The five publicly funded facilities serve an estimated catchment of 2 050 000 million people and included two university teaching hospitals out of four in the country and three district hospitals out of 42. No provincial referral or specialised hospitals were included. The four semiprivate specialised rehabilitation centres funded by the NGOs or FBOs, in the country were all included. These institutions serve an estimated catchment of 430 000 people (Table 1a). Four of the nine facilities were in urban areas (Table 1b). The population estimates of the catchment area are those who would need rehabilitation services in the area and do not consider the number who could pay for the services. Therefore, the number of people who can access semiprivate services is considerably less.

**TABLE 1b:** Distribution of rehabilitation services by geographical area and type of health facility.

Geographical area	Type of facility	Total no†. of facilities in Rwanda	Sample for the current study	Facilities sampled	No† of staff			Catchment area	Rehab Staff per 10 000 in catchment
					PTs†	P&Os†	OTs†		
Urban	Tertiary National referral university teaching hospitals	4	2	Kigali University Teaching Hospital	9	4	0	750 000	0.2
				Rwanda Military Hospital	12	0	2	750 000	0.2
	District hospitals	10	1	Masaka Hospital	3	0	0	200 000	0.1
	Specialised hospitals	2	1	Inkurunziza Orthopedic Hospital	6	2	0	100 000	0.8
	Total	16	4	-	30	6	2	1 800 000	0.2
Rural	District hospitals	28	2	Kabgayi Hospital	2	0	0	200 000	0.1
				Murunda Hospital	2	0	0	150 000	0.1
	Semiprivate rehab centres	4	3	Gahini Rehab Centre	9	3	5	150 000	1.1
				Rilima Rehab Centre	7	1	0	80 000	1.0
				Gatagara Rehab Centre	13	6	1	100 000	2.0
	Total	32	5	-	33	10	6	680 000	1.0
**Total (urban & rural)**	-	**48**	**9**	**-**	**63**	**16**	**8**	**2 480 000**	**0.4**

No., number; MOH, Ministry of Health; HI, Humanity & Inclusion; PTs, physiotherapists; P&Os, prosthetists and orthotists; OTs, occupational therapists; catchment area: the area from which patients at the public hospital are drawn considers only the adult population and private facilities, the proportion within the area who can pay the required fees.

† , This is an estimate of those who would need rehabilitation services in the area and does not consider the number who could pay for the services. Therefore, the number of people who can access semiprivate services is considerably less.

Rehabilitation staff at the nine health facilities include 63 PTs, 16 P&Os and 8 OTs, serving both children and adults. The availability of rehabilitation personnel ranged from 2 PTs at district hospitals to 0 OTs at public hospitals. Whilst we had 13 PTs, 6 P&Os and 5 OTs at semiprivate specialised rehabilitation centres primarily funded by NGOs or FBOs, based on catchment areas, publicly funded facilities have an average of 0.2 rehabilitation staff per 10 000 people, and semiprivately funded facilities have an average of 1.2 rehabilitation personnel per 10 000 people (Table 1a). The availability of rehabilitation personnel differed significantly by location of the facility, with urban facilities having an average of 0.2 rehabilitation staff per 10 000 people compared to 1.0 rehabilitation staff per 10 000 people at rural facilities (Table 1b).

### Description of services received by respondents

A total of 213 participants were recruited, 87.0% from publicly funded hospitals (*n* = 186) and 13% (*n* = 27) from semiprivately funded facilities. Most participants received PT (*n* = 190 [89.0%]), with much fewer receiving P&O (*n* = 24 [11.0%]) or OT (*n* = 3 [1.4%]), and 8 (4.0%) received more than one service (Table 2a). A similar pattern was seen at the public hospitals where most participants received PT (*n* = 175 [95.0%]), with few receiving P&O (*n* = 10 [5.0%]) and OT (*n* = 2, 1.1%) and 2 (1.1%) receiving more than one service. At semiprivate facilities, half of the participants received PT and half received P&O (PT *n* = 15 [56.0%]; P&O *n* = 14 [52.0%]), with just two (7.0%) receiving both. Only one (4.0%) participant received OT. In urban settings, the majority of participants received PT (*n* = 154 [93.0%]), few received P&O (*n* = 12 [7.0%]) and two (1.2%) received OT (Table 2b). In rural areas, most participants received PT (*n* = 36 [77.0%]), one-quarter of participants received P&O (*n* = 12 [25.0%]) and one (2.0%) participant received OT.

**TABLE 2a:** Distribution of participants, by funding source, type of health facility visited and rehabilitation services received.

Funding source	Level of facility	No. of participants per facility *n* = 213		Name of facility	No. of participants per service		
		*n*	%		PT	P&O	OT
Public (some private funding but primarily government)	Tertiary university teaching hospital	76	41	Kigali University Teaching Hospital	66	10	0
		62	34	Rwanda Military Hospital	62	0	2
	District hospitals	20	11	Masaka Hospital	20	0	0
		21	11	Kabgayi Hospital	21	0	0
		7	3	Murunda Hospital	7	0	0
	Total	186	87	-	175	10	2
Semiprivate (some government funding but primarily private)	Specialised hospital	8	14	Inkurunziza Orthopedic Hospital	6	2	0
	Semiprivate rehab centres	15	57	HVP Gatagara Orthopedic and Rehab Centre	8	9	1
		3	11	Gahini Rehab Centre	1	2	0
		1	4	Rilima Orthopedic and Rehab Centre	0	1	0
	Total	27	13	-	15	14	1
**Total (public and semiprivate)**	-	**213**	**100**	**-**	**190**	**24**	**3**

No., number; rehab, rehabilitation; PT, physiotherapy; P&O, prosthetics and orthotics; OT, occupational therapy.

**TABLE 2b:** Distribution of participants, by geographical area, type of health facility visited and rehabilitation services received.

Geographical area	Type of facility	No. of participants per facility *n* = 213		Name of facility	No. of participants per service		
		*n*	%		PT	P&O	OT
Urban	Tertiary university teaching hospital	76	46	Kigali University Teaching Hospital	66	10	0
		62	37	Rwanda Military Hospital	62	0	2
	District hospitals	20	12	Masaka Hospital	20	0	0
	Specialised hospital	8	5	Inkurunziza Orthopedic Hospital	6	2	0
	Total	166	78	-	154	12	2
Rural	District hospitals	21	45	Kabgayi Hospital	20	0	0
		7	15	Murunda Hospital	7	0	0
	Semiprivate rehab centres	15	32	HVP Gatagara Orthopedic and Rehab Centre	8	9	1
		3	6	Gahini Rehab Centre	1	2	0
		1	2	Rilima Orthopedic and Rehab Centre	0	1	0
	Total	47	22	-	36	12	1
**Total**	**-**	**213**	**100**	**-**	**190**	**24**	**3**

No., number; rehab, rehabilitation; PT, physiotherapy; P&O, prosthetics and orthotics; OT, occupational therapy.

### Description of respondents

There were more men than women (60% vs. 40%) in our sample and more of our participants were unable to work than able to work (51% vs. 27%). The only difference in demographic characteristics for those in public facilities compared to semiprivate facilities was that there were more women (44% vs. 5%), and fewer people were employed (28% vs. 33%) (Table 3a). When comparing participants at urban versus rural settings, urban participants tended to be older (43% vs. 32%), separated or divorced (16% vs. 2%) and either unemployed or in a temporary job (25% vs. 17%) (Table 3b).

**TABLE 3a:** *P*-values for demographic characteristics by source of funding (public versus semiprivate).

Variable	Public (*n* = 186)		Semiprivate (*n* = 27)		*p*
	*n*	%	*N*	%	
**Sex**	-	-	-	-	0.011
Male	105	56	22	81	-
Female	81	44	5	19	-
**Age (years)**	-	-	-	-	0.198
18–< 30	42	23	10	37	
30–45	64	34	10	37	
> 45	80	43	7	26	
**Marital status**	-	-	-	-	0.246
Single or unmarried	51	27	12	44	-
Married and living together	101	54	14	52	-
Widowed	27	15	0	0	-
Separated or divorced	7	4	1	4	-
**Level of education**	-	-	-	-	0.484
None	24	13	3	11	-
Primary education	76	41	7	26	-
Secondary education and vocational training	57	31	11	41	-
Tertiary education (A1, A0, MSc, PhD)	29	15	6	22	-
**Employment status**	-	-	-	-	0.006
Unemployed	27	15	2	7	-
Permanent job	32	17	8	30	-
Temporary job	20	11	1	4	-
Student	9	5	4	15	-
Unable to work	97	52	12	44	-
**Type of disability**	-	-	-	-	0.968
Physical	177	95	27	100	-
Mental	1	0.5	0	-	-
Multiple	6	3	0	-	-
Others	2	1	0	-	-
**Having health insurance**	-	-	-	-	0.536
Yes	175	94	24	89	-
No	11	6	3	11	-
**Location of facility**	-	-	-	-	0.000
Urban	158	85	8	30	-
Rural	28	15	19	70	-

Note: The table presents data for only those who responded yes to the question. The variable ‘other’ is not included because it is not specified in the data.

**TABLE 3b:** *P*-values for demographic characteristics by source of funding (public versus semiprivate)

Variable	Urban (*n* = 166)		Rural (*n* = 47)		*p*
	*n*	%	*N*	%	
**Sex**	-	-	-	-	0.818
Male	97	58	30	64	-
Female	69	42	17	36	-
**Age (years)**	-	-	-	-	0.012
18–< 30	37	22	15	32	-
30–45	57	34	17	36	-
> 45	72	43	15	32	-
**Marital status**	-	-	-	-	0.008
Single or unmarried	43	26	20	43	-
Married and living together	90	54	25	53	-
Widowed	7	4	1	2	-
Separated or divorced	26	16	1	2	-
**Level of education**	-	-	-	-	0.448
None	19	11	8	17	
Primary education	64	39	19	40	-
Secondary education and vocational training	55	33	13	28	-
Tertiary education (A1, A0, MSc, PhD)	28	17	7	15	-
**Employment status**	-	-	-	-	0.041
Unemployed	24	14	5	11	-
Permanent job	30	18	10	21	-
Temporary job	18	11	3	6	-
Student	9	5	4	9	-
Unable to work	84	51	25	53	-
**Type of disability**	-	-	-	-	0.000
Physical	161	97	43	91	-
Mental	0	0	1	2	-
Multiple impairment	5	3	1	2	-
Others	0	0	2	4	-
**Having health insurance**	-	-	-	-	0.559
Yes	156	94	43	91	-
No	10	6	4	9	-

### Facilitators and barriers to rehabilitation use

**FIGURE 2:**
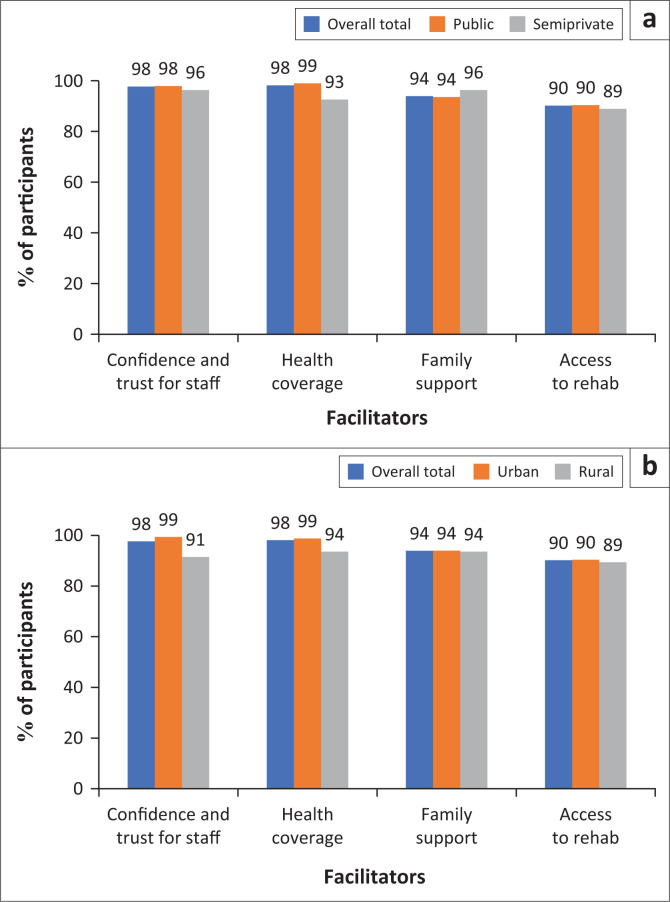
(a) Facilitators to rehabilitation use facility funding source where participants were recruited (public: *n* = 186; semiprivate: *n*: 27; (b) Facilitators to rehabilitation use by participants’ residence (urban: *n* = 166, rural: *n* = 47).

**FIGURE 3:**
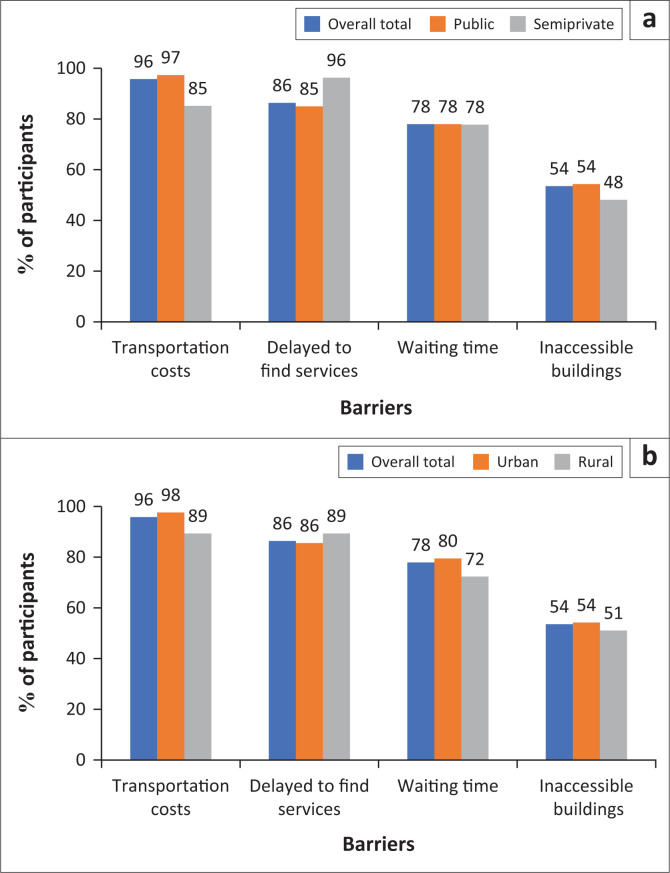
(a) Barriers to rehabilitation use by facility funding source where participants were recruited (public: *n* = 186, semiprivate: *n*: 27; (b) Barriers to rehabilitation use by participants’ residence (urban: *n* = 166, rural: *n* = 47).

Over 85% of participants considered the following as facilitators to rehabilitation service use: confidence and trust in staff, health coverage, family support and easily accessible services, which did not differ by facility except that patients at semiprivately funded facilities reported more family support than those from public facilities (96% vs. 78%) (Figure 2). The most common barriers to rehabilitation service use reported by over 60% of participants were transportation costs, delays in finding services, long waiting times and inaccessible buildings. Transportation cost was reported more by patients at public facilities than at semiprivate facilities (97% vs. 85%) (Figure 3).

## Discussion

With help from other international agencies, the Rwandan MoH published a report on a Situation Assessment of Rehabilitation in the Republic of Rwanda because of the recognition of the need to better understand rehabilitation service in Rwanda. This report highlights the lack of rehabilitation personnel, such as speech therapists, rehabilitation physicians and nurses (Ministry of Health-Rwanda 2021). This report also reveals that semiprivate funded rehabilitation centres are more equipped with resources than facilities funded by the public but falls short of understanding who is accessing rehabilitation services and why. Our data expand on the information from the Situation Assessment of Rehabilitation in the Republic of Rwanda report and other studies (Rhoda et al. 2015; Urimubenshi & Rhoda 2011) to describe resources at both public and semiprivate funded facilities in urban and rural settings and to describe who is accessing rehabilitation services. We also describe some patients’ facilitators and barriers to attending rehabilitation.

Our analysis determined that semiprivate facilities had six times more human resources than publicly funded facilities. Facilities in rural areas (predominantly semiprivately funded) had five times more human resources than those in urban areas. People accessed rehabilitation services primarily for physical disability concerns. Interestingly, the facilitators and barriers to rehabilitation services were similar across facilities, regardless of the funding source or geographic location. Our analysis highlights the most crucial facilitators for accessing rehabilitation, including patients’ confidence and trust in staff, family support and health coverage. At the same time, participants indicated transportation costs, waiting time and inaccessible buildings as some of the significant barriers.

It is well known that rehabilitation services are limited in low- and middle-income countries, which makes it imperative to use the existing resources where most needed. We demonstrated that semiprimarily funded facilities had six times more human resources than publicly funded facilities and that rural areas (predominantly semiprivately funded) had five times more human resources than those facilities in urban areas. However, it is unclear whether this distribution of resources reflects the proportion of Rwandans living with disabilities in each of these areas. Whilst rural (primarily semiprivate) facilities are better staffed, they are more difficult to access because patients must pay for services. Thus, the catchment population for the semiprivate facilities is likely much smaller, meaning that staff proportions are likely much higher for semiprivate facilities when considering people’s ability to pay. Whilst this increases the staffing ratios for those who can pay, it negatively affects the services available to those who cannot pay. Whilst numerically it seems that rural areas are better served, there are many additional considerations that suggest this may not be true. In addition to the issue of access because of payment, there is also the issue of distance to travel and costs associated with long-distance travel that may further affect access amongst the population in rural areas.

We demonstrated significant differences in some of the demographic characteristics of those who attended public versus semiprivate facilities in urban and rural settings; however, we do not know where the difference lies within these characteristics. We know that participants access rehabilitation services for physical disability concerns. However, it is unclear whether the needs of Rwandans with disability are primarily physical or whether the use of services is driven by the available services. Physiotherapists were the primary rehabilitation personnel available at all facilities, whilst P&Os were available at five facilities, of which one was publicly funded. Occupational therapists were available at one public facility and two semiprivate facilities, and there were no SLT services or physicians and nurses providing rehabilitation services. Whilst there is a clear need for rehabilitation services that address physical disabilities, it is unclear if other rehabilitation needs are not being addressed. Understanding where most Rwandans with disabilities reside and their needs is critical to optimally distribute services; however, with the available funding structure, it is unlikely that redistribution will solve the issues of access to services. In addition to better distribution, implementing the MoH’s new employment health structure proposed in 2020 will increase the overall number of rehabilitation professionals, particularly in areas with no rehabilitation services (Official Gazette 2020). Physiotherapists will be hired at PHCs, P&Os at district hospitals, OTs and SLTs employed at the referral and provincial hospitals for the first time. However, the implementation of this new structure is taking longer. The Situation Assessment of Rehabilitation in the Republic of Rwanda report emphasises the need to immediately implement the MoH’s new employment structure (Ministry of Health-Rwanda 2021). The Rwandan government is also exploring whether subsidies can be provided so that those with only universal health coverage can access semiprivately funded specialised centres. Whilst these government initiatives are positive, implementation could take time. Therefore, we suggest strategies to address the rehabilitation issues discussed without additional cost or burden to the system.

It is not surprising that most participants reported long waiting times as a significant barrier to attending rehabilitation. Even though there are six times more rehabilitation professionals at semiprivate facilities than public facilities, more than 70% of participants attending semiprivate facilities still noted waiting times as a significant barrier. This experience is similar to that of people seeking rehabilitation services in other low- and middle-income countries (Bright et al. 2018; Mlenzana et al. 2013; Ntamo, Buso & Longo-Mbenza 2013; Scheffler & Mash 2019).

Whilst the Rwandan universal healthcare coverage provides 90% – 100% (depending on the households’ economic situation) financial support for rehabilitation services at public facilities and semiprivate facilities, patients pay 100%; issues such as long waiting times and transportation costs remain significant barriers to accessing services. This is similar to issues noted in other low- and middle-income countries including South Africa and Nigeria (Bright et al. 2018; Igwesi-Chidobe 2012; Scheffler & Mash 2019). There is a critical need to identify strategies to capitalise on available resources to augment rehabilitation interventions without additional cost or burden to the system.

To decrease waiting times for those in most need, we need to develop triage referral protocols to provide faster services. This protocol could use a decision tree approach to help physicians identify those in the highest need, assign a priority rating and determine what care could likely be provided by a family member or a caregiver (Hobbs et al. 2010). Patients who need professionals’ interventions could then take the priority rating to a rehabilitation facility where one or two spots are held weekly to see priority referrals quickly. The University of Rwanda, the national associations of rehabilitation healthcare professionals, physicians and nurses could lead initiatives to develop triage protocols and decision trees to facilitate the process (Hobbs et al. 2010). Developing triage protocols could be implemented at the referral stage to prevent a constant flow of patients, long waiting lists and time. In addition, community-based interventions provided by families, community health workers (CHWs) and lay personnel might offer a sustainable alternative or complement to rehabilitation provided by health professionals (e.g., PTs) and provide an accessible and affordable approach with the potential to address the rehabilitation needs in the community in Rwanda.

Home or community-based interventions could also be developed to address the rehabilitation needs. The WHO proposed ‘task shifting’ more than a decade ago to supplement healthcare provision in low- and middle-income countries (World Health Organization 2008). More recently, ‘task sharing’ was suggested as an alternative to task shifting, when the responsibility for care provision cannot be completely assumed by those trained, skilled and credentialed to perform the tasks (Anand et al. 2019).

In addition to using the existing rehabilitation services more efficiently, we could supplement rehabilitation services by training existing healthcare providers on key aspects of rehabilitation. For example, rehabilitation professionals can offer appropriate and relevant training to nurses at PHCs, where rehabilitation services are nonexistent. The need to train nurses at PHCs was also recommended in the Situation Assessment of Rehabilitation in the Republic of Rwanda report (Ministry of Health-Rwanda 2021), and training initiatives could begin during professional training in university programmes.

Training non-healthcare professionals, such as family caregivers, CHWs and lay personnel, have been supported in previous studies conducted in low- and middle-income countries. For example, a study was conducted in Bangladesh (Rahman & Salek 2016) and Thailand (Pitthayapong et al. 2017) in which caregivers of patients with stroke were trained on incontinence care, bed positioning and activities of daily living (ADLs). The training provided to caregivers achieved positive results on patients’ outcomes, such as improved ADLs (Rahman & Salek 2016). Likewise, training CHWs in South Africa to provide rehabilitation interventions, such as transfers, bed mobility or positioning, has resulted in positive patient outcomes such as reduced pressure sores and improved mobility (Nesbit & Clark 2019).

Some studies have demonstrated the benefits of training caregivers and CHWs (Nesbit & Clark 2019; Magwood et al. 2020; Pitthayapong et al. 2017; Rahman & Salek 2016); however, additional research is needed to identify how to develop and evaluate these training programmes in Rwanda. Rehabilitation professionals could use existing community-based interventions and train CHWs to provide simple but effective physical rehabilitation interventions in the community (Anand et al. 2019; Dawson et al. 2014). The proposal to move rehabilitation from a therapist-led facility system is a significant shift; requiring buy-in from multiple stakeholders in multiple systems. It builds on the gains achieved through community-based rehabilitation (Lemmi et al. 2016; Mannan et al. 2012) and could create a community-sustained solution that could be funded at a fraction of the costs of a therapist-based facility solution for rehabilitation. We recognise this solution will take a buy on many levels and a revisioning of service provision.

Technology could also be used to augment rehabilitation services, particularly in countries with limited resources (Jesus et al. 2017; Jesus et al. 2019; Lincoln et al. 2014; Veitch et al. 2012). Low-cost technologies using mobile phones and telerehabilitation (where the Internet is available) could offer additional support for families and CHWs in Rwanda to provide rehabilitation interventions at home or in the community. For example, the University of Rwanda could introduce and support telerehabilitation interventions during outreach programmes. Telemedicine has been successfully developed and implemented in Rwanda (Roodenbeke et al. 2011). For example, mHealth programmes using mobile phones have increased maternal and child health services (Ruton et al. 2018) and increased access to antiretroviral drugs (Nsanzimana et al. 2012). These achievements have been possible because many Rwandans own a mobile phone. Telerehabilitation has provided cost-effective services in low- and middle-income countries (Fatoye et al. 2019; Sarfo et al. 2018) and could be an approach to increase access to rehabilitation services in Rwanda.

### Limitations of the study

We have undertaken a secondary data analysis to describe the people attending rehabilitation facilities but were limited to the data collected for the health economics study. Participants were purposively recruited, which could have biased their response to the data collectors, and thus did not provide the exact situation of the current rehabilitation issues for the Rwandan users. Although recruitment practices were limited from September to December 2018 and 3 days per week, we do or do not feel this limits the generalisability of our results because we recruited participants from both public and semiprivate facilities in urban and rural settings. A key consideration when interpreting our data is whether the sample was representative of those with disability in Rwanda and the facilities that they access. Our participants were identified when they attended a rehabilitation facility and therefore represent only those who can attend rehabilitation facilities (e.g., those who receive a referral, have a means of getting to the facility and get an appointment). Most data were obtained from urban and public facilities with an under-representation of rural and semiprivate facilities and no representation from referral or provincial hospitals. Therefore, we cannot state that similar patient profiles, barriers or facilitators are experienced in these settings. We were also limited in the number of variables we could include in a regression model if we had to look at predictors of being in a semiprivate or public hospital because we only had 27 people in the semiprivate hospitals. We also calculated the catchment for public and semiprivate facilities using estimates of the population in the area but did not have an idea of the proportion of the population who could afford versus those who cannot afford for semiprivate facilities.

Our study describes people who attended a facility, which is influenced by the services provided. We cannot generalise the findings regarding the type of disability because adults with disabilities use the available services (physiotherapy), not necessarily the services they need. Understanding the needs of persons with disabilities who require but cannot attend rehabilitation should be a future research priority. The survey only focused on patients with physical disabilities, which meant that our conclusion was only limited to this population. The survey’s primary purpose was to collect data on cost, which enabled us to only look at the quantity and not the quality of services. As a result, we had no data on the specific conditions (that resulted in physical disabilities), the number of sessions attended, needs and therapy outcomes of people attending facilities that provide rehabilitation services. This information would be beneficial to support the provision of appropriate rehabilitation care.

## Conclusions

The availability of rehabilitation services for Rwandan adults with physical disabilities is limited, with greater access to services in semiprivate compared to public facilities. Whilst family support help patients attend rehabilitation, transportation costs remain a significant barrier to people attending rehabilitation. Strategies to address these issues are urgently required and should complement the MoH solutions that will be implemented in the next years. These strategies can include developing triage protocols that could be implemented at the referral stage to prevent a constant flow of patients, long waiting lists and time. In addition, community-based interventions provided by families, CHWs, and lay personnel could extend the outreach of basic rehabilitation services to those with long-term health conditions and disabilities living in the community in Rwanda. This would require a major system shift that will require buy-in from multiple stakeholders in multiple systems but could be an efficient and sustainable solution. Finally, service provision could be augmented with the use of low-cost technologies using mobile phones and telerehabilitation (where the internet is available) and could offer additional support for families and CHWs in Rwanda to provide rehabilitation interventions at home or in the community.

## Figures and Tables

**FIGURE 1-A1:**
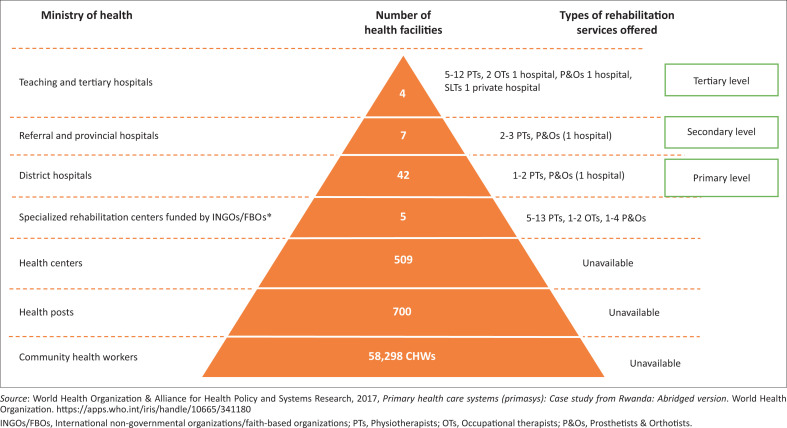
Rehabilitation Sector in the Health System. Adapted from the Organization chart of the health system in Rwanda.

## Footnotes

1
https://www.minaloc.gov.rw/fileadmin/user_upload/Minaloc/National_Policy_on_Disability_and_Inclusion_final.pdf

2
https://worldpopulationreview.com/countries/rwanda-population

## References

[CIT0001] Aenishänslin, J., Amara, A. & Magnusson, L., 2020, ‘Experiences accessing and using rehabilitation services for people with physical disabilities in Sierra Leone’, *Disability and Rehabilitation* 44(1), 34–43. 10.1080/09638288.2020.1755375 32352325

[CIT0002] Alleyne, G., Binagwaho, A., Haines, A., Jahan, S., Nugent, R., Rojhani, A. et al., 2013, ‘Embedding non-communicable diseases in the post-2015 development agenda’, *The Lancet* 381(9866), 566–574. 10.1016/S0140-6736(12)61806-6 23410606

[CIT0003] Anand, T.N., Joseph, L.M., Geetha, A.V., Prabhakaran, D. & Jeemon, P., 2019 ‘Task sharing with non-physician health-care workers for management of blood pressure in low-income and middle-income countries: A systematic review and meta-analysis’, *The Lancet Global Health* 7(6), e761–e771. 10.1016/S2214-109X(19)30077-4 31097278 PMC6527522

[CIT0004] Asiimwe-Kateera, B., Condo, J., Ndagijimana, A. & Kumar, S., 2015, ‘Analysis: Mobile health approaches to non-communicable diseases in Rwanda’, *Rwanda Journal* 2(1), 89. 10.4314/rjhs.v2i1.13F

[CIT0005] Baart, J. & Taaka, F., 2018, ‘Barriers to healthcare services for people with disabilities in developing countries: A literature review’, *Disability, CBR & Inclusive Development* 28(4), 26. 10.5463/dcid.v28i4.656

[CIT0006] Beguin, R. & Boisgillot, A., 2016, *How to conduct an economic analysis of physical and functional rehabilitation*, Humanity and Inclusion, Lyon.

[CIT0007] Boisgillot, A., 2020, ‘Towards inclusive health systems in low- and middle-income countries: Assessment of current and potential coverage of physical and functional rehabilitation care for people with disabilities’, PhD thesis, Dept. of Economics, University of Clermont Auvergne, pp. 1–317.

[CIT0008] Boisgillot, A. & Umuhoza, M.S., 2020, *A situational analysis of the financial access to rehabilitation services in Rwanda: Results of the IFAR diagnostic*, pp. 1–66, Humanity & Inclusion, Kigali.

[CIT0009] Bowker, L.K., Price, J.D. & Smith, S.C., 2006, ‘Chapter 4: Rehabilitation’, in World report on disability, pp. 95–133, World Health Organization, Geneva.

[CIT0010] Bright, T. & Kuper, H., 2018, ‘A systematic review of access to general healthcare services for people with disabilities in low and middle income countries’, *International Journal of Environmental Research and Public Health* 15(9), 1879. 10.3390/ijerph15091879 PMC6164773 30200250

[CIT0011] Bright, T., Wallace, S. & Kuper, H., 2018, ‘A systematic review of access to rehabilitation for people with disabilities in low- and middle-income countries’, *International Journal of Environmental Research and Public Health* 15(10), 2165. 10.3390/ijerph15102165 PMC6210163 30279358

[CIT0012] Canada Conference Board, 2017, *The market profile of physiotherapists in Canada*, viewed 30 October 2020, from https://physiotherapy.ca/sites/default/files/8695_profile-of-physiotherapists-in-canada_br.pdf

[CIT0013] Dawson, A.J., Buchan, J., Duffield, C., Homer, C.S.E. & Wijewardena, K., 2014, ‘Task shifting and sharing in maternal and reproductive health in low-income countries: A narrative synthesis of current evidence’, *Health Policy and Planning* 29(3), 396–408. 10.1093/heapol/czt026 23656700

[CIT0014] Fatoye, F., Gebrye, T., Fatoye, C. & Mbada, C.E., 2019, ‘Clinical and cost-effectiveness analysis of telerehabilitation intervention for people with nonspecific chronic low back pain (preprint)’, *JMIR mHealth and uHealth*. 10.2196/preprints.15375 PMC7381065 32357128

[CIT0015] Gimigliano, F. & Negrini, S., 2017, ‘The World Health Organization “rehabilitation 2030: A call for action”’, *European Journal of Physical and Rehabilitation Medicine* 53(2), 155–168. 10.23736/S1973-9087.17.04746-3 28382807

[CIT0016] Hamid, L.N., Kobusingye, O., Baine, S.O., Chrispus, M. & Bentley J. (2017) ‘Disability characteristics of community-based rehabilitation participants in Kayunga District, Uganda’, *Annals of Global Health* 83(3–4), 478–488. 10.1016/j.aogh.2017.10.006 29221520 PMC5728444

[CIT0017] Hategeka, C., Arsenault, C. & Kruk, M.E., 2020, ‘Temporal trends in coverage, quality and equity of maternal and child health services in Rwanda, 2000–2015’, *BMJ Global Health* 5(11), 1–10. 10.1136/bmjgh-2020-002768 PMC7668303 33187962

[CIT0018] Hobbs, J.A., Boysen, J.F., McGarry, K.A., Thompson, J.M. & Nordrum, J.T., 2010, ‘Development of a unique triage system for acute care physical therapy and occupational therapy services: An administrative case report’, *Physical Therapy* 90(10), 1519–1529. 10.2522/ptj.20090166 20688874

[CIT0019] Igwesi-Chidobe, C., 2012, ‘Obstacles to obtaining optimal physiotherapy services in a rural community in Southeastern Nigeria’, *Rehabilitation Research and Practice* 2012(2012), 1–8. 10.1155/2012/909675 PMC3437668 22973517

[CIT0020] Jesus, T.S., Arango-Lasprilla J.C., Kamalakannan, S.K. & Landry, M.D., 2021, ‘Growing physical rehabilitation needs in resource-poor world regions: Secondary, cross-regional analysis with data from the global burden of disease 2017’, *Disability and Rehabilitation* 44, 1–10. 10.3390/ijerph16060980 34086516

[CIT0021] Jesus, T.S., Landry, M.D., Dussault, G. & Fronteira, I., 2017, ‘Human resources for health (and rehabilitation): Six rehab-workforce challenges for the century’, *Human Resources for Health* 15(1), 1–12. 10.1080/09638288.2021.1933619 28114960 PMC5259954

[CIT0022] Jesus, T.S., Landry, M.D. & Hoenig, H., 2019, ‘Global need for physical rehabilitation: Systematic analysis from the global burden of disease study 2017’, *International Journal of Environmental Research and Public Health* 16(6), 980. 10.33137/cpoj.v1i2.31371 PMC6466363 30893793

[CIT0023] Lemaire, E.D., Supan, T.J. & Ortiz, M., 2018, ‘Global standards for prosthetics and orthotics’, *Canadian Prosthetics & Orthotics Journal* 1(2), 1–8. 10.33137/cpoj.v1i2.31371

[CIT0024] Lemmi, V., Blanchet, K., Gibson, L.J., Suresh, K.K., Rath, S., Hartley, S. et al., 2016, ‘Community-based rehabilitation for people with physical and mental disabilities in low- ana middle-income countries: a systematic review and meta-analysis. *Journal of Development Effectiveness* 8(3), 368–387. 10.1080/19439342.2016.1157623

[CIT0025] Lincoln, M., Hines, M., Fairweather, C., Ramsden, R. & Martinovich, J., 2014, ‘Multiple stakeholders perspectives on teletherapy delivery of speech pathology services in rural schools: A preliminary, qualitative investigation’, *International Journal of Telerehabilitation* 6(2), 65–74. 10.5195/ijt.2014.6155 25945230 PMC4353008

[CIT0026] Loeb, M., 2013, ‘Disability statistics: An integral but missing (and misunderstood) component of development work’, *Nordic Journal of Human Rights* 31(3), 306–324. 10.18261/ISSN1891-814X-2013-03-03 26925181 PMC4766593

[CIT0027] Magwood, G.S., Nichols, M., Jenkins, C., Logan, A., Qanungo, S., Zigbuo-Wenzler, E. et al., 2020, ‘Community-based interventions for stroke provided by nurses and community health workers: A review of the literature’, *Journal of Neuroscience Nursing* 52(4), 152–159. 10.1097/JNN.0000000000000512 PMC7337158 32341258

[CIT0028] Manini, T., 2012, ‘Development of physical disability in older adults’, *Current Aging Sciencee* 4(3), 184–191. 10.2174/1874609811104030184 PMC3868456 21529321

[CIT0029] Mannan, H., Boostrom, C., MacLachlan, M., McAuliffe, E., Khasnabis, C. & Neeru, G., 2012, ‘A systematic review of the effectiveness of alternative cadres in community based rehabilitation’, *Human Resources for Health* 10(20), 1–8. 10.1186/1478-4491-10-20 22888953 PMC3465230

[CIT0030] Ministry of Justice-Rwanda, 2009, *Codes and Laws of Rwanda, Ministry of Justice. Law n° 01/2007 of 20/01/2007 Relating to Protection of Disabled Persons in General*, Rwanda-Ministry of Justice, Kigali.

[CIT0031] Ministry of Health, 2018, *Fourth health sector strategic plan 2018–2024*, viewed 15 July 2020, from https://www.childrenandaids.org/sites/default/files/2018-05/Rwanda_NatHealthSectorPlan_2018-2024.pdf.

[CIT0032] Ministry of Health-Rwanda, 2021, ‘A situation assessment of rehabilitation in Republic of Rwanda’, *Prepared for Rwanda Ministry of Health with support from the United States Agency for International Development*, ICRC and the World Health Organization, Kigali.

[CIT0033] M’Kumbuzi, V.R.P., Sagahutu, J.-B., Kagwiza, J., Urimubenshi, G. & Mostert-Wentzel, K. (2014) ‘The emerging pattern of disability in Rwanda’, *Disability and Rehabilitation* 36(6), 472–478. 10.3109/09638288.2013.798361 23738617

[CIT0034] Mlenzana, N.B., Frantz, J.M., Rhoda, A.J. & Eide, A.H., 2013, ‘Barriers to and facilitators of rehabilitation services for people with physical disabilities: A systematic review’, *African Journal of Disability* 2(1), 1–6. 10.4102/ajod.v2i1.22 PMC5442576 28729982

[CIT0035] Morris, S., Fawcett, G., Brisebois, L. & Hughes, J. 2018, ‘Canadians survey on disabilities: A demographic, employment and income profile, 2017’, *Statistics Canada* 1–25, viewed 15 January 2021, from https://www150.statcan.gc.ca/n1/en/daily-quotidien/181128/dq181128a-eng.pdf?st=ehaI_KDQ.

[CIT0036] National Institute of Statistics of Rwanda & Ministry of Finance and Economic Planning-Rwanda, 2014, *Fourth Population and Housing Census, Rwanda, 2012 Thematic Report Socio-economic characteristics of persons with disabilities*, National Institute of Statistics of Rwanda, Kigali.

[CIT0037] National Institute of Statistics of Rwanda Kigali, Rwanda Ministry of Health Kigali, Rwanda & The DHS Program ICF, 2021, *Rwanda Demographic and Health Survey 2019-20 Final Report*, National Institute of Statistics of Rwanda and ICF, Kigali and Rockville, MD.

[CIT0038] Nesbit, K.C. & Clark, A., 2019, ‘Rehabilitation training for community health workers: A five-year study’, *International Journal of Health Promotion and Education* 57(1), 3–12. 10.1080/14635240.2018.1538808

[CIT0039] Ng, L.C. & Harerimana, B., 2016, ‘Mental health care in post-genocide Rwanda: Evaluation of a program specializing in posttraumatic stress disorder and substance abuse’, *Global Mental Health* 3(18), 1–11. 10.1017/gmh.2016.12 PMC5012309 27610238

[CIT0040] Nsanzimana, S., Ruton, H., Lowrance, D.W. & Cishahayo, S., 2012, ‘Cell phone-based and internet-based monitoring and evaluation of the national antiretroviral treatment program during rapid scale-up in Rwanda: TRACnet, 2004–2010’, *Journal of Acquired Immune Deficiency Syndromes* 59(2), 17–23. 10.1097/QAI.0b013e31823e2278 22067668

[CIT0041] Ntamo, N., Buso, D. & Longo-Mbenza, B., 2013, ‘Factors affecting poor attendance for outpatient physiotherapy by patients discharged from Mthatha general hospital with a stroke’, *SA Journal of PhySiotherapy* 69(3), 19–24. 10.4102/sajp.v69i3.29

[CIT0042] Official Gazette-Rwanda, 2020, *Prime Minister’s Instructions determining organisational structure, salaries and fringe benefits for employees of Referral Hospitals, Provincial Hospitals, District Hospitals, Specialised Hospitals Medicalised Heath Centres and Health Centres: Official Gazette n° Special of 01 September 2020*, Laws.Africa, Kigali.

[CIT0043] Pitthayapong, S., Thiangtam, W., Powwattana, A., Leelacharas, S. & Waters, C.M., 2017, ‘A community based program for family caregivers for post stroke survivors in Thailand’, *Asian Nursing Research* 11(2), 150–157. 10.1016/j.anr.2017.05.009 28688501

[CIT0044] Prynn, J.E. & Kuper, H., 2019, ‘Perspectives on disability and non-communicable diseases in low-and middle-income countries, with a focus on stroke and dementia’, *International Journal of Environmental Research and Public Health* 16(18), 3488. 10.3390/ijerph16183488 PMC6766001 31546803

[CIT0045] Rahman, M.S. & Salek, A.K.M., 2016, ‘Training of caregiver for home care management of stroke survivor at low resource setting’, *Bangabandhu Sheikh Mujib Medical University Journal* 9(4), 193. 10.3329/bsmmuj.v9i4.30158

[CIT0046] Rhoda, A., Cunningham, N., Azaria, S. & Urimubenshi, G., 2015, ‘Provision of inpatient rehabilitation and challenges experienced with participation post discharge: Quantitative and qualitative inquiry of African stroke patients’, *BMC Health Services Research* 15(1), 1–9. 10.1186/s12913-015-1057-z 26412081 PMC4584463

[CIT0047] Roodenbeke, D.E., Lucas, S., Rouzaut, A. & Bana, F., 2011, *Outreach services as a strategy to increase access to health workers in remote and rural areas*, World Health Organization, Geneva. 26269879

[CIT0048] Rugema, L., Krantz, G., Mogren, I., Ntaganira, J. & Persson, M., 2015, ‘“A constant struggle to receive mental health care”: Health care professionals’ acquired experience of barriers to mental health care services in Rwanda’, *BMC Psychiatry* 15(1), 1–9. 10.1186/s12888-015-0699-z 26672596 PMC4682265

[CIT0049] Ruton, H., Musabyimana A., Gaju E., Berhe, A., Grépin, K.A., Ngenzi, J. et al., 2018, ‘The impact of an mHealth monitoring system on health care utilization by mothers and children: An evaluation using routine health information in Rwanda’, *Health Policy and Planning* 33(8), 920–927. 10.1093/heapol/czy066 30169638 PMC6172419

[CIT0050] Sarfo, F.S., Adusei, N., Ampofo, M., Kpeme, F.K. & Ovbiagele, B., 2018, ‘Pilot of a tele-rehab intervention to improve outcomes after stroke in Ghana: A feasibility and user satisfaction study’, *Journal of Neurological Sciences* 15(387), 94–97. 10.1016/j.jns.2018.01.039 PMC5868410 29571880

[CIT0051] Scheffler, E. & Mash, R., 2019, ‘Surviving a stroke in South Africa: Outcomes of home-based care in a low-resource rural setting’, *Topics in Stroke Rehabilitation* 26(6), 423–434. 10.1080/10749357.2019.1623473 31169468

[CIT0052] Smith, S., Kayiteshonga, Y., Misago, C.N., Iyamuremye, J.D., d’Arc Dusabeyezu, J., Mohand, A.A. et al., 2017, ‘Integrating mental health care into primary care: The case of one rural district in Rwanda’, *Intervention* 15(2), 136–150. 10.1097/WTF.0000000000000148

[CIT0053] Statistics South Africa, 2014, *Census 2011: Profile of persons with disabilities in South Africa*, Statistics South Africa, Pretoria.

[CIT0054] Tapela, N.M., Bukhman, G., Ngoga, G., Kwan, G.F., Mutabazi, F., Dusabeyezu, S. et al., 2015, ‘Treatment of non-communicabledisease in rural resource-constrained settings: A comprehensive, integrated, nurse-led care model at public facilities in Rwanda’, *The Lancet Global Health* 3, 36. 10.1016/S2214-109X(15)70155-5

[CIT0055] Urimubenshi, G. & Rhoda, A., 2011, ‘Environmental barriers experienced by stroke patients in Musanze district in Rwanda: A descriptive qualitative study’, *African Health Sciences* 11(3), 398–405, viewed 06 February 2021, from http://www.ncbi.nlm.nih.gov/pubmed/22275930%0Ahttp://www.pubmedcentral.nih.gov/articlerender.fcgi?artid=PMC3261026. 22275930 PMC3261026

[CIT0056] Veitch, C., Dew, A., Bulkeley, K., Lincoln, M., Bundy, A., Gallego, G. et al., 2012, ‘Issues affecting therapist workforce and service delivery in the disability sector in rural and remote New South Wales, Australia: Perspectives of policymakers, managers and senior therapists’, *Rural and Remote Health* 12(2), 1–12. 10.22605/RRH1903 22681246

[CIT0057] Vollset, S.E., Goren, E., Yuan, C-W. & Cao, J., 2020, ‘Fertility, mortality, migration, and population scenarios for 195 countries and territories from 2017 to 2100: A forecasting analysis for the Global Burden of Disease Study’, *The Lancet* 396(10258), 1285–1306. 10.1016/S0140-6736(20)30677-2 PMC7561721 32679112

[CIT0058] Wade, D.T., 2020, ‘What is rehabilitation? An empirical investigation leading to an evidence-based description’, *Clinical Rehabilitation* 34(5), 571–583. 10.1177/0269215520905112 32037876 PMC7350200

[CIT0059] Wanyana, D., Wong, R. & Hakizimana, D., 2021, ‘Rapid assessment on the utilization of maternal and child health services during COVID-19 in Rwanda’, *Public Health Action* I1(1), 12–21. 10.5588/pha.20.0057 PMC7987248 33777716

[CIT0060] WCPT, 2019a, *World conderation for physical therapy: Profile for physical therapists in South Africa*, pp. 1–5, viewed 30 October 2020, from http://documents1.worldbank.org/curated/en/688761571934946384/pdf/Doing-Business-2020-Comparing-Business-Regulation-in-190-Economies.pdf.

[CIT0061] WCPT, 2019b, *World conferation for physical therapy: Profile of physical therapists in 2019*, 1–9, viewed 30 October 2020, from https://www.wcpt.org/sites/wcpt.org/files/files/cds/reports/2018/150066.pdf.

[CIT0062] WHO, 2008, *Task shifting- global recommendations & guidelines*, World Health Organization, Geneva.

[CIT0063] World Health Organization (WHO), 2011, *World report on disability*.

[CIT0064] World Health Organization (WHO), 2014, *WHO global disability action plan 2014–2021*, World Health Organisation, Geneva, pp. 1–5.

[CIT0065] World Health Organization (WHO), 2017, ‘The need to scale up rehabilitation’, *Rehabilitation*, 1–9, viewed from https://apps.who.int/iris/handle/10665/331210

[CIT0066] World Health Organization & Alliance for Health Policy and Systems Research, 2017, *Primary health care systems (primasys): Case study from Rwanda: Abridged version*. World Health Organization. https://apps.who.int/iris/handle/10665/341180.

[CIT0067] Zziwa, S., Babikako, H., Kwesiga, D., Kobusingye, O., Bentley, J.A., Oporia, F. et al., 2019, ‘Prevalence and factors associated with utilization of rehabilitation services among people with physical disabilities in Kampala, Uganda, A descriptive cross sectional study’, *BMC Public Health* 19(1), 1–11. 10.1186/s12889-019-8076-3 31881994 PMC6935194

